# Prevalence and factors associated with failed induction of labor in Worabe Comprehensive Specialized Hospital, Southern Ethiopia

**DOI:** 10.1371/journal.pone.0263371

**Published:** 2022-01-28

**Authors:** Muhdin Mohammed, Rewda Oumer, Fatuma Mohammed, Fantahun Walle, Hassen Mosa, Ritbano Ahmed, Shamill Eanga

**Affiliations:** 1 Department of Nursing, Kibet Primary Hospital, Kibet, Ethiopia; 2 Department of Nursing, Worabe Comprehensive Specialized Hospital, Worabe, Ethiopia; 3 Department of Nursing, College of Medicine and Health Sciences, Wolkite University, Wolkite, Ethiopia; 4 Department of Midwifery, College of Medicine and Health Sciences, Wachemo University, Hossana, Ethiopia; 5 Department of Anesthesia, College of Medicine and Health Sciences, Wolkite University, Wolkite, Ethiopia; Debre Tabor University, ETHIOPIA

## Abstract

**Background:**

Induction of labor is one of the most used obstetric procedures in the world. It is performed in around 20% of all pregnancies. Failed induction of labor, on the other hand, has been associated with poorer mother and newborn health outcomes. Besides, there is a scarcity of data on the current burden and drivers. Therefore, this study aimed to assess the prevalence and factors associated with failed induction in Worabe Comprehensive Specialized Hospital, Southern Ethiopia.

**Methods:**

A retrospective cross-sectional study was conducted on medical records of mothers who delivered through induction of labor during September 1^st^, 2018 to August 30^th^, 2020. The samples were collected using a systematic sampling technique. The data was extracted using a checklist. Data were entered into EpiData (version 3.1) and analyzed using SPSS (version 24). Multivariable logistic regression analyses were used to decide the association of explanatory variables with the outcome variable. Odds ratio with their 95% CI were calculated to identify the presence and strength of an association. A p-value of < 0.05 was used to declare statistical significance.

**Results:**

In this study, the prevalence of failed induction was observed to be 22.2%. The associated factors included rural residence (AOR = 5.7, 95% CI: 3.12–11.02), primiparity (AOR = 8.4, 95% CI: 2.72–22.36) and unfavourable bishop score (AOR = 5.9, 95% CI: 4.52–16.12).

**Conclusions:**

In comparison to the rate reported in developed countries, the study area had a high rate of failed induction. Being rural residence, primiparity and unfavourable bishop score were the associated factors of failed induction. Therefore, to reduce of the rate of failed induction, health care practitioners should analyze cervical status (using Bishop Score) to decide the possibility of successful induction, with a focus on associated factors like parity.

## Introduction

Induction of labor is the artificial stimulation of uterine contractions before the spontaneous onset of true labor at 28 or more weeks of gestation to achieve vaginal delivery [1]. Induction could be emergency or planned. Generally, induction of labor should be conducted when the advantages to the fetus or mother outweigh the dangers of enduring the pregnancy. The rates of induction of labor differ from one area to another area with progressive increase to approximately doubling of frequency in some of the developed countries [2, 3]. For example, about 20% of total deliveries in the United States and the United Kingdom are induced, with some organizations going as high as 40%, while 11.4% is recorded in Latin America [2, 4], and an average of 4.4% in Africa [5].

There is a controversy in the definition of failed induction of labor. However, it is usually diagnosed when there has been no cervical change or descent of the presenting part after 6–8 hours of labor, or contraction of 3 in 10 minute has not been attained[1, 6]. Failed induction has been associated with maternal and perinatal complications [7], as well as a higher incidence of instrumental and caesarean births [8].

In Ethiopia, just a few studies on failed induction of labor have been performed. Furthermore, it has a regional variation in its rate. In Addis Ababa, Jimma, and Hawassa, for example, it was 25.4, 21.4, and 17.3%, respectively [9–11].

A multifaceted range of factors has been associated with failed induction of labor comprising maternal factors like primiparity, maternal age, poor bishop score, fetal factors like birth weight and gestational age [10–17]. Moreover, the obtained results regarding failed induction of labor are not conclusive and have shown discrepancies among populations and ethnic-geographical groups. However, the Ethiopian government has made several attempts to improve access to facility-based maternal and child health care facilities in order to minimize maternal morbidity and mortality. In spite of this, in 2016 the maternal mortality rate in the country was found to be high (412 deaths per 100,000 live births) [18].

Generally, failed induction of labor is a cause for concern because the reason is obscure to health care providers. Moreover, there is a paucity of research on the degree and predisposing factors of failed induction in the study area. Therefore, the goal of this study was to assess the prevalence and factors associated with failed induction of labor in Worabe Comprehensive Specialized Hospital. The outcomes of induction of labor at the health facility level will be helped as a catalog to track the extent and frequent indications in order to improve the quality of care. In addition, the findings can be used to provide evidence-based information to mothers who are considering inducing labor.

## Methods and materials

A retrospective cross-sectional study was conducted by reviewing medical records of induced deliveries duringSeptember 1^st^, 2018 to August 30^th^, 2020 at the Worabe Comprehensive Specialized Hospital, Silte Zone, Southern Ethiopia. Worabe Town is located 172 kilometers south of Ethiopia’s capital, Addis Ababa. The hospital offers a full range of obstetric services, including skilled delivery, and has a high volume of maternity patients. During the study period there were 8000 deliveries, 1400 of them were induced deliveries. The data was collected from November 01 to November 30, 2020.

The source population comprised all women who underwent induction of labor after 28 weeks of gestation at the Worabe Comprehensive Specialized Hospital from September 1^st^, 2018 to August 30^th^, 2020.The study population encompassed systematically selected women who underwent induction of labor after 28weeks of gestation in Worabe Comprehensive Specialized Hospital from September 1^st^, 2018 to August 30^th^, 2020. However, maternal records which had incomplete registering were excluded from this study.

### Eligibility criteria

#### Inclusion criteria

All enrolled women who had induction of labor after 28 weeks of gestation in Worabe Comprehensive Specialized Hospital maternity ward from September 1^st^, 2018 to August 30^th^, 2020.

#### Exclusion criteria

All enrolled women undergoing labor induction without complete documentation.

The sample size was computed using a single population proportion formula with the following assumptions: a 95% confidence interval, 5% margin of error and 21.4% proportion of failed induction which was taken from a study conducted at the Jimma Specialized Hospital [10]. After adjusting 10% for incomplete medical records, the final sample size was 284. A systematic random sampling technique was employed to recruit the charts every fifth interval. The first chart was selected randomly by a lottery method on the initial day of the data collection period.

### Measurements

#### Failed induction

In this study, the meaning of failed induction of labor was attained from the mothers’ medical chart which was identified by the data collectors. Failed induction of labor was considered as “yes” (when mothers’ have diagnosed as experienced failed induction of labor in their medical chart) or “no”.

#### Bishop score

The Bishop Score predicts the possibility of vaginal delivery after induction of labor.

#### Favourable Bishop score

Those Bishop score having a value of greater than six.

#### Unfavourable Bishop score

Those Bishop score having a value of less than or equal to six.

#### Post-term pregnancy

A pregnancy that persists for 42 weeks or more from the onset of the last menstrual period or 40 weeks of gestation from the time of conception.

#### Elective induction

Labor induction is supposed to be elective, when it is under taken for the reason of convenience and in the absence of any maternal and fetal condition that justifies delivery.

#### Emergency induction

Is carried out when the indication incurs urgent and serious complication.

### Data collection procedure

Data were collected through review of medical records using a checklist which was developed after appraising of various related literatures [9–11].The checklist was designed to collect information on the sociodemographic characteristics, obstetric factors, induction related factors, and fetal condition. Three diploma midwives who can speak both Siltigna and Amharic were hired to collect data, and one BSc midwife assisted in supervising the data collectors.

To ensure the quality of data, the tool was translated first to Siltigna (a local language) and then translated back to English to check its consistency. The reliability of the checklist was tested by carrying out a pretest on 5% of the sample size in the Worabe health centre before the actual study period. Next to the pretest, understand ability, unambiguousness, and organization of the checklist were checked. After, applying the reliability test, 0.87Cronbach’s alpha value was gained. The validity of the checklist was confirmed by the proper application of validity criteria. Also, training was given for data collectors and supervisors on the content of the checklist, the goals of the study and the data collection procedure. Further, the supervisors and the investigators firmly monitored the day-to-day data collection process during the pre-test and the actual data collection period. The filled checklist was collected and signed by the supervisor after their completeness was confirmed by checking for any missing items and logicality.

### Data processing and analysis

Data were entered into the EpiData software (version 3.1) and analyzed using SPSS software (version 24). The dependent variable was “failed induction of labor” (yes = 1; no = 0).Initially, bivariate logistic regression was performed for the selection of candidate variables into multivariable logistic regression. In binary logistic regression, the variables with a *p*-value < 0.25 were transferred to the multivariable logistic regression model. It was conducted to discover the independent associated factors of the outcome variable and control probable confounders. Odds ratio with their 95% confidence intervals was calculated to identify the existence and strength of association, while statistical significance was stated at a *p*-value < 0.05.

## Results

### Socio-demographic characteristics

A 284 chart of mother’s document has evaluated in the study period. The record retrieval rate was100%.The mean age of the mothers was 26.4 (standard deviation ± 5.7) years. The majority of mothers were married 280(98.6%), 157(55.2%) had followed a primary level of education, and152 (53.4%) were residing in urban areas (**Table 1**).

**Table 1:** Sociodemographic characteristics of mothers at Worabe Comprehensive Specialized Hospital, Southern Ethiopia, 2020.

Variables	Frequency	Percent
**Age in years**		
<20	80	28.2
20–34	180	63.3
≥35	24	8.5
**Residence**		
Urban	132	46.5
Rural	152	53.5
**Marital status**		
Married	280	98.6
Unmarried	4	1.4
**Educational status**		
No formal education	25	8.8
Primary	157	55.2
Secondary	82	28.9
College and above	20	7.1

### Obstetric characteristics

One hundred fifty five (54.6%) participants were multiparous, 220 (77.5%) had four or more ANC visits. The majority of the participants, 221 (77.8%) gave birth through vaginally.On physical examination, 126(44.4%) were found to have unfavourable bishop score, while 158(55.6%) had a favourable bishop score. Post-term pregnancy was the most common indication for induction of labor101 (35.6%), followed by PROM 71(25%), and hypertensive disorders 58 (20.4%).The overall prevalence of failed induction was observed to be 63 (22.2%) (**Table 2**).

**Table 2:** Obstetrics and physical examination findings characteristics of the participants at Worabe Comprehensive Specialized Hospital, Southern Ethiopia, 2020.

Variables	Frequency	Percent
**Parity**		
Primiparous	129	45.4
Multiparous	155	54.6
**Number of ANC visit**		
Four and above visits	220	77.5
Less than four visit	64	22.5
**Mode of delivery**		
Vaginal delivery	221	77.8
Caesarean section	63	22.2
**Gestational age**		
Term	174	61.3
Post-term	110	38.7
**Types of induction**		
Planned/Elective	35	12.3
Emergency	249	87.7
**Bishop score**		
Unfavorable	126	44.4
Favorable	158	55.6
**Indications for induction of labor**		
Post-term pregnancy	101	35.6
Prolonged rupture of membranes	71	25
Hypertensive disorders of pregnancy	58	20.4
Maternal medical complication	13	4.6
Non-reassuring antepartum fetal testing	41	14.4
**Cervical ripening**		
Yes	126	44.4
No	158	55.6
**Methods of induction**		
Oxytocin	213	75
Misoprostol	71	25
**5**^**th**^ **Minutes APGAR score**		
<7	54	19
≥7	230	81
**Birth weight**		
2.5–4 kg	229	80.6
>4 kg	55	19.4
**Outcomes of induction**		
Successful Induction	221	77.8
Failed Induction	63	22.2

### Ethics approval and consent to participate

Ethical clearance was obtained from Wolkite University research and community service vice president office before data collection. Official letter was written to the Worabe Comprehensive Specialized Hospital to get permission. Then, formal letter was written from the medical director of Worabe Comprehensive Specialized Hospital to the record office. Lastly, the hospital record office gave us permission to collect the necessary data. However, informed consent from respondents was not required since the nature of the study was extracting and analyzing the existing data, which posed minimal risks to the respondents. Thus, the ethics committee waived the need for informed consent. Nonetheless, data collectors maintained confidentiality through eliminating names or any other personal identifiers from data collection sheets and reports.

### Factors associated with failed induction of labor

The outcome of the multivariable logistic regression analysis showed that, rural residence, primiparity and unfavourable bishop score were associated factors of failure in labor induction.

The odds of experiencing failed induction were 6 times more likely in women who have an unfavourable bishop score as compared to those women who have a favourable bishop score(AOR = 6, 95% CI: 4.52–16.12).

Similarly, the odds of having failed induction were observed to be 8.4 times higher among primiparous women in comparison to those reported for multiparous women(AOR = 8.4, 95% CI: 2.72–22.36).

Furthermore, women who resided in rural areas had 5.7 times more odds of experiencing failed induction of labor than those women who resided in urban areas(AOR = 5.7, 95% CI: 3.12–11.02) (**Table 3**).

**Table 3:** Factors associated with failed induction of labor at Worabe Comprehensive Specialized Hospital, South Ethiopia, 2020.

Variables	Failed induction		COR(95% CI)	AOR(95% CI)
	Yes	No		
**Residence**				
Rural	53(18.7)	10(3.5)	6.5 (3.16–13.49)*	5.7(3.12–11.02)**
Urban	99(34.8)	122(43)	Reference	Reference
**Bishop score**				
Unfavorable	49(17.2)	77(27.2)	6.7(3.39–12.60)*	6(4.52–16.12)**
Favorable	14(4.9)	144(50.7)	Reference	Reference
**Parity**				
Primiparous	52(18.3)	77(27.2)	8.8(4.36–17.92)*	8.4(2.72–22.36)**
Multiparous	11(3.8)	144(50.7)	Reference	Reference
**Birth weight**				
>4 kg	45(15.9)	10(3.6)	5.9(3.62–8.75)*	4(0.8–6.72)
2.5–4 kg	99(45.8)	130(46.7)	Reference	Reference

*p ≤ 0.25,

**p < 0.05.

## Discussion

The present study has examined the frequency of failed induction of labor and its associated factors in the Worabe Comprehensive Specialized Hospital. In this research, the rate of failed induction of labor was found to be 22.2%.This prevalence is higher than observed in studies conducted in Jimma, Hawassa and Pakistan, which were 21.4, 17, and 18.1%, respectively [10, 11, 13]. The higher prevalence may be attributed to the fact that the study’s hospital was a referral hospital. Furthermore, this disparity may be an indication of the failure of national maternal care service policies.

In this study, the probability of failure in induction of labor was significantly associated with being primiparous. This may be because primiparous women differ from multiparous women in terms of cervical effacement prior to induction of labor and their response to cervical ripening methods. Commonly, a primiparous woman’s uterus is less receptive to oxytocin. Besides, primiparous women have no previous labor experience, as a result achieving the appropriate rate of cervical collagen fiber dissolution become more difficult with respect to multiparous women who have a previous labor experience. This finding is consistent with the results of different studies conducted in Jimma [10], Hawassa [11], Pakistan [13] and Israel [19].

Bishops score was found to be the associated factors of failed induction. This may be because the cervix’s condition (ripening) at the time of induction is a crucial factor in the effectiveness of induced labor. The existence of subjectivity in the evaluation of bishop score may also explain the discrepancy. This finding is consistent with studies undertaken in Jimma, Hawassa and Pakistan [10, 11, 13].

This study also reveals that, the likelihood of failure in induction of labor was significantly associated with being rural residents. The explanation for this may be that women in rural areas are more likely than women in urban areas to be subject to difficult labor, which raises the likelihood of a failed induction. Furthermore, women who live in urban areas have greater access to health care than women who live in rural areas, which may help prevent abnormal labor.This finding is in line with studies undertaken in Dessie(Ethiopia) and Tanzania [20, 21].

The strengths of this study comprise the fact that study participants were selected using the probability sampling method to ensure the representativeness of the study, and different approaches were used to maintain the quality of data. However, the retrospective nature of the study and the lack of certain critical variables due to improper and/or non-recording of such variables are obvious drawbacks of cross-sectional studies. Additionally, since bishop scoring is subjective, there may be some inter-personal differences.

## Conclusion

In comparison to the rate reported in developed countries, the study area had a high rate of failed labor induction. After controlling for possible confounders, being rural, primiparity and having an unfavourable bishop score were the determinants of failed induction of labor. Therefore, to reduce of the rate of failed induction, health care practitioners should analyze cervical status (using the Bishop score) to decide the possibility of successful induction, with a focus on associated factors like parity. Besides, large scale longitudinal studies should be conducted to explore more about the wide-ranging contributing factors of failed induction of labor.

## Supporting information

S1 File Questionnaire. (DOCX)

S2 File SPSS. (SAV)

S1 Checklist (DOCX)

## Abbreviations

AOR: Adjusted odds ratio
APGAR: Appearance pulse grimace activity and respiration
CI: Confidence interval
COR: Crude odds ratio
SPSS: Statistical package for social science
