# Supplementary material for: Prevalence and factors associated with failed induction of labor in Worabe Comprehensive Specialized Hospital, Southern Ethiopia
Source: PLoS One. 2022 Jan 28;17(1):e0263371. doi: 10.1371/journal.pone.0263371 (PMC8797230; doi:10.1371/journal.pone.0263371)
Supplement: S1 File — (DOCX) [file pone.0263371.s001.docx]

| Part I. Socio-demographic data | | |
| --- | --- | --- |
| No | Question | Answers and codes |
| 1 | Age of the women in years | ….…………… |
| 2 | Residence | 1. Urban 2. Rural |
| Part II. Obstetric data | | |
| 3 | Gestational age | --------in weeks |
| 4 | Para |  |
| 5 | ANC visit | 1. Yes 2. No |
| Part III. Induction of labor | | |
| 6 | Was the induction of labor failed | 1. Yes 2. No |
| 7 | The length of time from induction to delivery | -----------hours |
| 8 | Types of the induction | 1. Planned 2. Emergency |
| 9 | Bishop score at time of admission | ---------------- |
| 10 | Indication(s) for induction,of labor | 1. Postdated pregnancy 2. Premature rapture of membrane 3. Hypertensive disorders 4. Maternal medical complications (eg,Diabetes,renal,chronic pulmonarydisease,chronic hypertension ) 5. Fetalcompromise(eg,Isoimmunization,oligohydramnios,NRFHR,IUGR) 6. Fetal death 7. Antepartum hemorrhage 8. Chorioamnionitis 9. Others specify |
| 11 | Membranes already ruptured before induction? | 1. Yes  2. No |
| 12 | Meconium stained amniotic fluid present following induction of labor? | 1.Yes  2. No |
| 13 | Method used for induction of labor | 1. Oxytocin infusion only 2. Oxytocin with prior cervical repining 3. Misoprostol 4. Artificial ROM 5. Foley catheter |
| 14 | Birth weight in kg | ---------------------- |
| 15 | Apgar score at 1 and 5 min | Score at 1 min _________  Score at 5 min________ |
| 16 | Status of newborn | 1. alive   2.dead |
